# Understanding health literacy of deaf persons with hypertension in South Korea: A cross-sectional study

**DOI:** 10.1371/journal.pone.0294765

**Published:** 2023-11-27

**Authors:** Gi Won Choi, Sun Ju Chang, Hee Jung Kim, Ha Na Jeong

**Affiliations:** 1 College of Nursing, Seoul National University, Seoul, Republic of Korea; 2 Center for Human-Caring Nurse Leaders for the Future by Brain Korea 21 (BK 21) Four Project, College of Nursing, Seoul National University, Seoul, Republic of Korea; 3 College of Nursing and The Research Institute of Nursing Science, Seoul National University, Seoul, Republic of Korea; Kasturba Medical College Mangalore, Manipal Academy of Higher Education, INDIA

## Abstract

**Background:**

Health literacy is strongly associated with health inequality among persons with deafness, and hypertension (HTN) is the most prevalent chronic disease among persons with deafness in South Korea. Despite its importance, research regarding the health literacy levels of persons with deafness with HTN in South Korea is lacking. This study aimed to comprehensively assess the health literacy levels of persons with deafness with HTN in South Korea, including linguistic, functional, and internet health literacy.

**Methods:**

In this descriptive cross-sectional study, 95 persons with deafness with HTN were recruited through facilities associated with the deaf community. From August 2022 to February 2023, data were collected through face-to-face surveys attended by a sign language interpreter and online surveys. The data were analyzed using descriptive statistics and Spearman’s correlation.

**Results:**

Approximately 62.1% of the participants exhibited a linguistic health literacy level corresponding to less than that of middle school students, and the total percentage correct of functional health literacy was 17.9%. Each domain of internet health literacy was low. Significant correlations were found between some aspects of health literacy.

**Conclusions:**

The study’s findings highlight the low health literacy levels across various facets among persons with deafness with HTN in South Korea. Based on these findings, several strategies are suggested for developing HTN self-management interventions for persons with deafness. This study contributes to the foundational understanding of health literacy among persons with deafness with HTN in South Korea and provides valuable insights and guidance for developing HTN self-management interventions.

## Introduction

The World Health Organization estimates that approximately 460 million people have hearing loss worldwide; the number is projected to reach 700 million, or one in ten people, by 2050 due to global population growth and aging [1, 2]. Among persons with hearing loss, those with difficulty communicating due to severe hearing loss are called "deaf." [3] Previous research has highlighted significant health inequalities experienced by persons with deafness, including limited access to healthcare services and health information compared to the hearing population [4]. According to the PROGRESS PLUS framework, health inequality can be influenced by place of residence, race, occupation, gender, religion, education, socioeconomic status, social capital, age, disability, and sexual orientation [5]. Interestingly, previous studies have identified two primary causes of health inequality among persons with deafness: communication difficulties resulting from language barriers associated with their disability, as mentioned in the PROGRESS PLUS framework, and inadequate levels of health literacy [6, 7].

Health literacy refers to "people’s knowledge, motivation and competences to access, understand, appraise, and apply health information in order to make judgments and take decisions in everyday life concerning healthcare, disease prevention and health promotion to maintain or improve quality of life during the life course." [8] Low levels of health literacy induce poor disease-related knowledge levels, and adversely affect self-management, which can be detrimental for patients with chronic diseases [9].

Hypertension (HTN) is a well-known chronic disease associated with an increased risk of premature mortality [10]. Previous studies have mentioned that HTN can potentially affect the microcirculation of the inner ear and act as a risk factor for hearing loss [11, 12]. This establishes a significant link between HTN and deafness. In South Korea, HTN is highly prevalent among the deaf population, accounting for 63.9% [13]. This rate is considerably higher than the general population, where the prevalence is reported as 28.1% for adults aged ≥ 19 and 62.3% for individuals aged ≥ 65 [14]. HTN’s high prevalence among persons with deafness can be attributed to the characteristic age distribution within the South Korean deaf population, with a sharp increase in deaf cases after age 50 [13]. Furthermore, considering the likelihood of developing HTN increases with age [15], HTN is a crucial chronic condition among persons with deafness in South Korea.

In the case of HTN, self-management, such as healthy diet, weight control, and regular exercise, plays a crucial role in effectively controlling blood pressure (BP) [16]. However, persons with deafness with HTN may face challenges in managing their condition due to limited health literacy. HTN self-management education can be beneficial in such cases [17]. Moreover, tailoring interventions to the specific health literacy levels of persons with deafness can mitigate the impact of limited health literacy and enhance the effectiveness of interventions [18].

First, it is necessary to understand the health literacy level of persons with deafness with HTN [9]. Various tools are available for measuring health literacy, and depending on the tool used, more focused health literacy can be assessed. For instance, the Test of Functional Health Literacy in Adults (TOFHLA) [19], one of the representative measurement tools, can identify functional health literacy by determining whether individuals can read and understand health-related data and take appropriate actions [20]. Functional health literacy has fluid cognitive characteristics, as health literacy tends to decrease with age [21, 22]. Conversely, another widely used tool, the Rapid Estimate of Adult Literacy in Medicine (REALM), assesses linguistic health literacy by measuring understanding of medical terms [23]. Linguistic health literacy has a stable characteristic over time, which slightly differs from functional health literacy [21, 22]. Additionally, tools like the eHealth Literacy Scale (eHEALS) [24] and the Digital Health Technology Literacy Assessment Questionnaire (DHTL-AQ) [25] are available to measure health literacy related to the internet or digital platforms. Combining these tools with different characteristics enables a more comprehensive understanding of the participants’ health literacy.

While a few studies have assessed the level of health literacy in the general deaf population, they relied on sign language versions of a single health literacy tool [26], a single question regarding confidence in writing the medical forms [27], or qualitative approaches [28, 29]. As a result, the understanding of health literacy has been limited, focusing on specific aspects and lacking a comprehensive perspective. Furthermore, previous studies have primarily focused on non-Asian countries, such as the United States, Australia, and Zambia, leading to a limited understanding of the health literacy levels among persons with deafness in Asian countries. In South Korea, a previous study utilizing national statistical data focused on individuals aged ≥ 19 and revealed that approximately half of the general population possessed adequate health literacy levels [30]. However, there is a lack of research specifically examining health literacy among persons with deafness in South Korea.

Therefore, this study aims to comprehensively assess the health literacy level of persons with deafness with HTN in South Korea before development of HTN self-management programs specifically tailored for this population. Various tools were utilized to measure different aspects of health literacy, including linguistic, functional, and Internet health literacy.

## Materials and methods

### Study design

This is a descriptive cross-sectional design study to identify the levels of linguistic, functional, and internet health literacy of persons with deafness with HTN in South Korea.

### Participants

This study targeted persons with deafness with HTN using facilities associated with the deaf community, such as institutions affiliated with the Korea Association of the Deaf, welfare centers, and churches. The inclusion criteria for the study were: (a) age ≥ 18 years, (b) registered as deaf in accordance with the Korean Disabled Persons Welfare Act, (c) use of sign language or lip-reading, and (d) diagnosed with HTN by a physician for at least 6 months. The six-month time period was selected based on the findings of a previous study targeting patients with chronic diseases; the duration was set at six months, the time it takes for patients to recognize the disease and develop self-care skills [31]. The exclusion criteria were (a) diagnosis of mental illness (e.g., alcoholism, schizophrenia, drug addiction) that hindered participation in the study and (b) refusal to participate in the study. The required number of study participants was calculated using the G-power 3.1.9.7 program. Based on Cohen [32], with a medium effect size (f) of .30, a significance level of .05, and a power of .80, using a two-tailed test, 84 people were required. Considering a dropout rate of 20%, we planned to recruit 101 persons; however, only 99 people were ultimately recruited. Of those, 95 data sets were included in the final analysis, because it was discovered that four participants had been diagnosed with HTN for less than six months and were therefore excluded.

### Data collection

Data collection was conducted from August 2022 to February 2023. Before initiation, we contacted the individual in charge of each facility related to persons with deafness to explain the purpose of the study and requested their assistance. As some participants may have felt apprehensive about face-to-face surveys due to COVID-19, face-to-face and online surveys were conducted to enable participants to choose their preferred method. For the face-to-face survey, a sign language interpreter was present at the participants’ request to explain the research and consent procedure. After obtaining written consent for participation in the study, a questionnaire was provided. Since reading comprehension was also being measured [33], sign language interpretation was not provided for the functional health literacy questionnaire. However, sign language interpretation was provided as needed for the linguistic and internet health literacy assessments. If an online survey was desired, a link to the online survey was sent to the participants. On the first page of the link, there were explanations regarding participation in the study and questions to determine whether they met the requirements for participation and were willing to voluntarily participate in the study. If they checked "Yes" to the above questions, the survey page was made available.

### Measurements

#### Demographic and disease-related characteristics

The demographic characteristics included gender, age, education level, subjective economic status, living arrangement, and religion. Subjective economic status was assessed by asking about difficulties in providing food, clothing, and shelter, with response options ranging from "very difficult" to "difficult" or "not difficult." Living arrangement was determined by asking about the individuals’ cohabitants. For disease-related characteristics, HTN-related information (duration of HTN, medication use status and duration, BP, height, weight, smoking, drinking and comorbidities) and deafness-related information (degree, duration, cause, sign language/lip-reading use status and duration) were measured.

#### Linguistic health literacy

Linguistic health literacy was measured using the Korean Health Literacy Assessment Tool-2 (KHLAT-2) [34], developed based on the REALM [23]. As REALM was measured by the ability to read words, but in the case of Korean, it is easy to read even if the person does not know the meaning of the words, KHLAT-2 allows respondents to choose between "knowing exactly" or "not knowing exactly" for 66 words and calculates a total score of 66 points by counting the number of questions answered as "knowing exactly." [34] Depending on the total score and REALM’s classification, 0–3rd grade (0–18 points) is below the level of 3rd grade elementary school for the general population, 4–6th grade (19–44 points) being the level of 4–6th grade elementary school for the general population, and 7–8th grade (45–60 points) being middle school 1–2nd grade level for the general population, and more than 9th grade (61–66 points) mean level of 3rd grade or higher in middle school for general population [35]. At the time of development, Cronbach’s alpha was 0.97 [34]. In this study, Cronbach’s alpha was 0.98.

#### Functional health literacy

Functional health literacy was assessed using the Short Form of the Korean Functional Health Literacy (S-KFHLT), which was abbreviated by Kim [36]. The Korean Functional Health Literacy Test (KFHLT), the original version of the S-KFHLT, was modified according to Korean culture [37], based on the TOFHLA [19]. The S-KFHLT comprises two main domains: numeracy and reading comprehension. The numeracy domain contains 4 questions: “Check drug expiration date,” “Check blood glucose normality,” “Medications to be taken on an empty stomach: 1 h before meals,” and “Medications to be taken on an empty stomach: 2–3 h after meals.” The reading comprehension domain includes 4 questions: “Abdominal ultrasonography schedule: What to eat on the morning of the examination,” “Abdominal ultrasonography schedule: Inspection time,” “Consent for colorectal polyps’ resection: Things to be notified of before the examination,” and “Consent for colorectal polyps’ resection: Complications related to examination.” For each question, 1 point is given if the answer is correct and 0 points for incorrect answers and is calculated out of 8 points. The higher the score, the better the functional health literacy. In the study by Kim [36], Cronbach’s alpha was 0.84. The Cronbach’s alpha in this study was 0.89.

#### Internet health literacy

Internet health literacy was measured using the DHTL-AQ tool [25]. This tool is composed of three domains corresponding to digital functional literacy (Information and communication technology (ICT) terms, ICT icons, application use) and one domain corresponding to digital critical literacy (evaluation of reliability and relevance of health information). The 11 questions involving ICT terms are given 1 point each if participants answer that they know and 0 points if they do not know, and the 9 questions regarding ICT icons are given 1 point if the icon and term are correctly matched, and 0 points if incorrect. The 9 questions about the use of the application are given 1 point for "Yes," and 0 points for the others, whereas 5 questions about digital critical literacy are given 1 point for “I agree" and 0 points for "I disagree," with a total of 34 points. Higher scores correspond to higher internet health literacy. At the time of development, Cronbach’s alpha was 0.95 [25], and in this study, it was found to be 0.96.

### Ethical considerations

This study was approved by the Institutional Review Board of the authors’ affiliations (Approval No. 2208/004-008). All researchers completed training in understanding persons with deafness through the Seoul Regional Health & Medical Center for Persons with Disabilities (North). Researchers provided sufficient explanations to the participants about the purpose and study procedure, the protection of human rights, and the guarantee of data anonymity. Moreover, during the face-to-face survey, the participants’ characteristics were considered, such as wearing a transparent mask that showed the shape of their lips or having a sign language interpreter present so that the participants did not feel uncomfortable during the study. The researchers also provided a comprehensive explanation of the study to the sign language interpreters, to enable them to convey accurate information to the participants. Additionally, to ensure consistency in interpreting the study information, only two sign language interpreters were utilized. Throughout the entire process, COVID-19 quarantine guidelines were followed.

### Statistical analysis

The participants’ characteristics and the measured variables were described using descriptive statistics such as frequency, percentage, mean, and standard deviation. Additionally, in the case of variables, the percentage awareness or percentage answered correctly for each item was calculated together according to the characteristics of the assessment tools. Normality was confirmed using the Kolmogorov–Smirnov test. Spearman’s correlation was conducted to analyze the correlation between variables, because each aspect of health literacy was not normally distributed. For statistical analysis, SPSS version 26.0 software (IBM Corp., Armonk, NY, USA) was used, and *p*-value < 0.05 was considered statistically significant.

## Results

### General characteristics of study participants

Table 1 summarizes the general characteristics of the 95 study participants; the information was primarily collected through face-to-face surveys (88.4%). The mean age of the participants was 64.81 ± 9.83 (range 34.00–85.00) years; more than half were females (64.2%) and high school graduates and above (51.6%). Furthermore, the majority lived with their families (71.6%) and were Christians (80.0%). Almost half responded that they were not in financial difficulties (47.4%). The mean duration of HTN was 10.99 ± 8.62 (range 0.75–40.00) years; most participants were taking medication (95.8%). According to the participants’ self-reported results, the mean systolic BP (SBP) was 138.27 ± 16.33 (range 105.00–190.00) mmHg, and the mean diastolic BP (DBP) was 90.14 ± 18.63 (range 50.00–141.00) mmHg. Approximately 90.5% responded that they knew their SBP, whereas 67.4% knew their DBP. Most participants knew their height (96.8%) and weight (97.9%), and approximately three-quarters had comorbid conditions apart from HTN (73.7%). For the deafness-related characteristics, most participants had severe deafness (93.7%), and the duration of deafness was 53.42 ± 17.98 (range 1.00–84.00) years. 75.8% of participants acquired deafness, while only 5.3% were congenital cases. Participants mainly used sign language (92.6%), and less than half used lip-reading (35.8%). The mean duration of sign language use was 46.86 ± 16.58 (range 6.00–73.00) years, and for lip-reading users, the mean duration of lip-reading use was 41.34 ± 21.51 (range 2.00–71.00) years.

**Table 1:** General characteristics of study participants (N = 95).

Variable	Categories	n (%) or Mean ± SD*
**Survey methods**	Face-to-face	84 (88.4)
	Online	11 (11.6)
**Demographic characteristics**		
Gender	Male	34 (35.8)
	Female	61 (64.2)
Age (year) (range 34.00–85.00)		64.81 ± 9.83
	< 60	29 (30.5)
	≥ 60	66 (69.5)
Education level	≤ Elementary	22 (23.2)
	Middle	24 (25.3)
	≥ High	49 (51.6)
Subjective economic status	Very difficult	9 (9.5)
	Difficult	41 (43.2)
	Not difficult	45 (47.4)
Living arrangement	Living alone	27 (28.4)
	Living with family	68 (71.6)
Religion	Christian	76 (80.0)
	Non-Christian	19 (20.0)
**Disease-related characteristics**		
Duration of HTN^†^ (year) (range 0.75–40.00)		10.99 ± 8.62
Use of HTN medication	Yes	91 (95.8)
	No	4 (4.2)
Duration of HTN medication (year) (range 0.08–32.00)		10.14 ± 8.06
Systolic BP^‡^ (mmHg)		138.27 ± 16.33
(range 105.00–190.00)	Know	86 (90.5)
	Do not know	9 (9.5)
Diastolic BP (mmHg)		90.14 ± 18.63
(range 50.00–141.00)	Know	64 (67.4)
	Do not know	31 (32.6)
Height	Know	92 (96.8)
	Do not know	3 (3.2)
Weight	Know	93 (97.9)
	Do not know	2 (2.1)
Smoking	Yes	3 (3.2)
	No	92 (96.8)
Drinking	Yes	25 (26.3)
	No	70 (73.7)
Comorbidity	Yes	70 (73.7)
	No	25 (26.3)
Degree of deafness	Severe	89 (93.7)
	Mild	6 (6.3)
Duration of deafness^§^ (range 1.00–84.00)		53.42 ± 17.98
Causes of deafness	Congenital	5 (5.3)
	Acquired^||^	72 (75.8)
	Unknown	18 (18.9)
Use sign language	Yes	88 (92.6)
	No	7 (7.4)
Duration of sign language use (year)^¶^ (range 6.00–73.00)		46.86 ± 16.58
Use lip-reading	Yes	34 (35.8)
	No	61 (64.2)
Duration of lip-reading use (year)^#^ (range 2.00–71.00)		41.34 ± 21.51

*Note*. *SD: Standard deviation, ^†^HTN: Hypertension, ^‡^BP: Blood pressure

^§^Duration of deafness was analyzed except for three people who said they did not know.

^||^Acquired causes were included infection, measles, nerve palsy, seizure, old age, otolithiasis etc.

^¶^Duration of sign language use was analyzed except for two people who said they did not know.

^#^Duration of lip-reading use was analyzed except for two people who said they did not know.

### The level of linguistic health literacy

The results presented in Table 2 indicate that the mean score for linguistic health literacy was 33.41 ± 21.25 (range 0.00–66.00), with an overall percentage awareness of 50.6%. Most participants scored in the 4–6th grade range (31.6%), followed by the 0–3rd grade range (30.5%). When examining each item, the highest awareness was observed for “Arthritis” (74.7%), “Fatigue” (72.6%), “Obesity” (68.4%), “After a meal,” “Constipation,” and “Nutrition” (67.4%), while the lowest awareness was observed for “Gonorrhea” (16.8%), “Incest” (17.9%), “Impetigo,” “Smear” (24.2%), and “Bowel” (29.5%).

**Table 2:** Linguistic health literacy of the study participants (N = 95).

Total	n (%)
0–3rd grade (0–18 points)	29 (30.5)
4–6th grade (19–44 points)	30 (31.6)
7–8th grade (45–60 points)	26 (27.4)
≥ 9th grade (61–66 points)	10 (10.5)
Mean ± SD* (range 0.00–66.00)	33.41 ± 21.25
Awareness percentage (%)	50.6
**Item**	**Percentage awareness (%)**
**Highest item**	
Arthritis	74.7
Fatigue	72.6
Obesity	68.4
After a meal	67.4
Constipation	67.4
Nutrition	67.4
**Lowest item**	
Gonorrhea	16.8
Incest	17.9
Impetigo	24.2
Smear	24.2
Bowel	29.5

*Note*. *SD: Standard deviation

### The level of functional health literacy

Table 3 shows the level of functional health literacy of the participants. The average score was 1.43 ± 2.31 (range 0.00–8.00), and the total percentage correct was 17.9%. Based on the domains, the mean score was 0.78 ± 1.32 (range 0.00–4.00) for numeracy and 0.65 ± 1.17 (range 0.00–4.00) for reading comprehension. The percentages correct were 19.5 and 16.3%, respectively. The item with the highest percentage correct in numeracy was “Check blood glucose normality” (25.3%), and the lowest was “Medications to be taken on an empty stomach: 2–3 h after meals” (14.7%). In reading comprehension, “Abdominal ultrasonography schedule: What to eat on the morning of the examination” (25.3%) was the highest, and “Consent for colorectal polyps’ resection: Things to be notified of before the examination,” “Consent for colorectal polyps’ resection: Complications related to examination” (9.5%) yielded the lowest percentages correct.

**Table 3:** Functional health literacy of the study participants (N = 95).

Item		Percentage correct (%)
**Numeracy**		19.5
1. Check drug expiration date		21.1
2. Check blood glucose normality		25.3
3–4. Medications to be taken on an empty stomach	3. 1 h before meals	16.8
	4. 2–3 h after meals	14.7
Mean ± SD* (range 0.00–4.00)		0.78 ± 1.32
**Reading comprehension**		16.3
5–6. Abdominal ultrasonography schedule	5. What to eat on the morning of the examination	25.3
	6. Inspection time	21.1
7–8. Consent for colorectal polyps’ resection	7. Things to be notified of before the examination	9.5
	8. Complications related to examination	9.5
Mean ± SD (range 0.00–4.00)		0.65 ± 1.17
**Total**		17.9
Mean ± SD (range 0.00–8.00)		1.43 ± 2.31

*Note*. *SD: Standard deviation

### The level of internet health literacy

The level of internet health literacy among the participants is shown in Table 4, with a total mean score of 8.72 ± 9.47 (range 0.00–34.00). The mean score of ICT terms was 1.16 ± 1.77 (range 0.00–5.00), and the percentage awareness was 10.5%. The term that participants responded they knew the most was “application” (27.4%), and the term least known was “wearable device” (1.1%). Meanwhile, the mean score of ICT icons was 2.86 ± 3.39 (range 0.00–9.00), and the percentage correct was 31.8%. “QR (Quick response) code” (45.3%) was the highest icon that participants correctly matched, and “download” (25.3%) was the lowest icon. The average score of the remaining digital critical literacy was 1.57 ± 2.79 (range 0.00–11.00), and application use was 3.13 ± 3.62 (range 0.00–9.00).

**Table 4:** Internet health literacy of the study participants (N = 95).

Domain	Item	Percentage awareness (%)	Domain	Item	Percentage correct (%)
ICT* **terms**		10.5	**ICT icons**		31.8
	Play store (app store)	20.0		URL	33.7
	Application	27.4		Search bar	34.7
	Update (synchronization)	22.1		Social media	27.4
	Search bar	17.9		Bluetooth	38.9
	Bluetooth	20.0		Download	25.3
	Web browser	4.2		Synchronization	33.7
	QR^†^ code	21.1		Voice assistant	42.1
	Chatbot (voice assistant)	9.5		Security file	31.6
	Wearable device	1.1		QR code	45.3
	Cloud	6.3	Mean ± SD (range 0.00–9.00)		2.86 ± 3.39
	Domain (URL)	7.4	**Application use**		
Mean ± SD^‡^ (range 0.00–5.00)		1.16 ± 1.77	Mean ± SD (range 0.00–9.00)		3.13 ± 3.62
**Digital critical literacy**			**Total**		
Mean ± SD (range 0.00–11.00)		1.57 ± 2.79	Mean ± SD (range 0.00–34.00)		8.72 ± 9.47

*Note*. *ICT: Information and communication technology

^†^QR: Quick Response

^‡^SD: Standard deviation

### Correlation between aspects of health literacy

Table 5 displays the findings of the Spearman correlation to determine the relationship between each aspect of health literacy. Linguistic health literacy showed a significant correlation only in digital critical literacy (rho = 0.262, *p* = 0.010) and application use (rho = 0.354, *p* < 0.001) in internet health literacy. In the case of functional health literacy and internet health literacy, a significant correlation was found between each domain, and the correlation coefficient (rho) ranged from 0.412 to 0.656. (*p* < 0.001).

**Table 5:** Correlation between aspects of health literacy (N = 95).

Variable		Linguistic	Functional		Internet			
			Numeracy	Reading comprehension	ICT terms	Digital critical literacy	ICT icons	Application use
**Linguistic**		1						
**Functional**	**Numeracy**	0.128 (0.216)	1					
	**Reading comprehension**	0.184 (0.074)	0.652*** (<0.001)	1				
**Internet**	**ICT**^†^ **terms**	0.092 (0.375)	0.568*** (<0.001)	0.535*** (<0.001)	1			
	**Digital critical literacy**	0.262* (0.010)	0.493*** (<0.001)	0.415*** (<0.001)	0.557*** (<0.001)	1		
	**ICT icons**	0.164 (0.112)	0.482*** (<0.001)	0.418*** (<0.001)	0.656*** (<0.001)	0.491*** (<0.001)	1	
	**Application use**	0.354*** (<0.001)	0.521*** (<0.001)	0.412*** (<0.001)	0.582*** (<0.001)	0.610*** (<0.001)	0.563*** (<0.001)	1

*Note*. *p < .05

**p < .01

***p < .001

^†^ICT: Information and communication technology

## Discussion

We aimed to conduct a comprehensive assessment of the health literacy level of persons with deafness with HTN in South Korea, and the level was generally identified as low. Based on these findings, several suggestions were provided on how to approach the planning of HTN self-management interventions for persons with deafness when considering their health literacy.

First, in linguistic health literacy, 62.1% of the participants had a level equivalent to less than that of middle school students for the general population, even though more than half of them completed high school or higher education. These findings agree with those of a previous study indicating that the health literacy of persons with deafness is at risk regardless of their education level [38]. Additionally, a previous study targeting individuals with spinal injury, in which more than half possessed education at junior college or above, revealed that only 24.7% of participants had a level equivalent to less than that of middle school students for the general population [39]. This suggests that the linguistic health literacy of persons with deafness with HTN is lower than that of people with spinal cord injury.

Upon examining the items of linguistic health literacy in this study, it was observed that commonly used items in daily life were highly recognized by the participants. Interestingly, among these items, arthritis, a disease name, had the highest recognition rate. This finding differs from a previous study that ranked arthritis 13^th^ among middle-aged general hospitalized patients [40]. This disparity may be attributed to the participants in our study predominantly use sign language, which involves hand and finger joints [41], and the age distribution of the participants, with over two-thirds being over 60 years old—a vulnerable group for arthritis [42]—suggesting a heightened interest in this condition. Conversely, items with lower awareness percentages were specialized medical terms that are less encountered in everyday life. Considering these results, it is advantageous to use simple words commonly encountered in daily life when designing HTN self-management interventions for persons with deafness. Additionally, if complicated medical terminology needs to be used, explaining the term using simple words in advance could be helpful. This approach can ultimately empower their linguistic health literacy and improve their understanding of the medical terms used in the interventions.

In the numeracy domain, one aspect of functional health literacy, percentage correct regarding medication-related questions, “Check drug expiration date,” “Medications to be taken on an empty stomach: 1 h before meals,” and “Medications to be taken on an empty stomach: 2–3 h after meals,” were ranged from 14.7% to 21.1%. These results were lower compared to a previous study involving older hearing people with an average age of 67.7 years, where the percentage ranged from 59.4% to 83.8% [43]. Moreover, a study primarily consisting of older people, similar to our study, revealed that persons with deafness exhibited lower levels of understanding regarding medication use compared to their hearing counterparts [44]. Considering that most of our participants were taking medication for HTN and 73.7% reported having a comorbid condition, there is a potential risk that they may be taking a combination of various drugs without proper knowledge of how to take them correctly. This finding aligns with previous research involving individuals who were deaf across various age groups, which also reported difficulties in comprehending the explanations provided on medication packaging [45]. Therefore, when developing HTN self-management interventions for persons with deafness, it is crucial to strengthen the content related to medication use. Additionally, it is important to evaluate their actual understanding rather than solely providing general drug guidelines.

The percentage of correct responses for questions related to consent for colorectal polyps’ resection, specifically "Things to be notified of before the examination" and "Complications related to the examination," were found to be the lowest among all functional health literacy items. This finding is consistent with a previous study focused on older hearing people [43]. The previous study mentioned that the difficulty in understanding could be attributed to the presence of complex concepts beyond the participants’ reading and writing abilities [43]. This explanation can also be applied to our participants. However, another potential reason could be the compositional aspect of the consent form, which consists of only long sentences. Unlike a previous study targeting hearing patients with chronic disease [46], there was no significant correlation between functional health literacy and linguistic health literacy in the present study. This implies that even when health-related materials use familiar words, it can still be challenging for persons with deafness to comprehend and act upon. According to a previous study on literacy among persons with deafness in South Korea, presenting information as noun or phrase units and using visual materials can increase the percentage correct [47]. Therefore, it is essential to consider appropriate expression methods that go beyond the use of familiar words, including the use of short phrases and visual materials when designing HTN self-management interventions for persons with deafness.

Looking at the overall score of internet health literacy, it was approximately 25% level, with digital critical literacy scoring even lower at around 15%. A previous study focusing on persons with deafness of various age groups mentioned their frequent reliance on the internet for obtaining health information [48]. However, considering that a vast amount of health information of varying quality exists on the internet [49] and the development of various health applications targeting persons with deafness [50], the importance of digital critical literacy becomes evident. The low level of digital critical literacy among the participants in this study suggests a high risk of exposure to incorrect health information, emphasizing the need for improvement in this area. Furthermore, this study revealed a significant positive correlation between digital critical literacy and functional health literacy. This finding aligns with previous research indicating a negative correlation between the evaluation and trust of online health information and low levels of functional health literacy [51]. In the process of health information processing, the step of "understanding" precedes the step of "evaluation" related to judgment [8]. Therefore, when establishing goals for HTN self-management interventions for persons with deafness, improving both functional health literacy and digital critical literacy together, can create a synergistic effect rather than solely focusing on improving digital critical literacy.

Another interesting aspect of internet health literacy is that the percentage awareness of ICT terms was lower than the percentage correct of ICT icons. Particularly, when considering items that were commonly asked in both domains, it was found that approximately half of the percentage correct of ICT icons for Bluetooth and QR codes appeared as awareness of ICT terms, whereas URL accounted for only one-fifth. This may be due to the widespread use of Bluetooth in IoT (Internet of Things) technology [52] and the prevalent use of QR codes during the COVID-19 pandemic for electronic access records and vaccination certificates [53]. QR codes were also utilized to connect sign language videos for persons with deafness [54]. In this context, the positive correlation between ICT terms, ICT icons, application use, and functional health literacy can be explained by a previous study indicating that an appropriate level of functional health literacy increases internet access [55]. As internet usage increases, users may become more familiar with the terms, icons, and applications commonly used online. Consequently, similar to linguistic health literacy, familiarity can be considered an important aspect of internet health literacy. Therefore, when developing HTN self-management interventions for persons with deafness using digital intervention methods, including the internet, it is advisable to first familiarize them with the digital environment and provide explanations to aid their understanding of related terms and icons. This is particularly crucial for the gradually increasing number of persons with deafness in the older age group.

This study possessed several limitations. First, most participants engaged in face-to-face surveys; however, caution is required in generalized interpretation as there may be potential differences depending on whether face-to-face or online surveys were conducted. Second, to maintain accuracy and consistency in data collection, we educated sign language interpreters about our research and utilized only two sign language interpreters. However, as the researcher did not directly communicate in sign language and instead interacted through a sign language interpreter, there could have been some distortion in the delivery of the research explanation and progress. Third, the participants in this study were recruited through convenience sampling, which may restrict the generalizability of the findings. In particular, as many participants were older individuals, the results of this study may be influenced by characteristics common to this age group. Additionally, the measured health literacy levels in this study may have been lower than actual levels because most participants had comorbid conditions known to lower health literacy [56]. Lastly, further research should explore the relationship between acquired deafness and health literacy, as acquired deafness could potentially be linked to infections or delayed treatment resulting from low health literacy. Nevertheless, to our knowledge, this is the first study to examine health literacy from multiple perspectives in persons with deafness with HTN in South Korea. The study provides valuable suggestions to consider when developing HTN self-management interventions for persons with deafness, making it an invaluable resource that can be extended and applied to future studies involving persons with deafness with chronic illness.

## Conclusions

This study was conducted to comprehensively evaluate the health literacy level of persons with deafness with HTN in South Korea. The findings revealed low levels of linguistic, functional, and internet health literacy among the participants, with significant correlations observed between certain aspects of health literacy. Based on these findings, several strategies have been proposed for the development of HTN self-management interventions for persons with deafness. These strategies include using simple words, incorporating short phrases and visual materials, enhancing medication-related content, improving functional health literacy and digital critical literacy simultaneously, and familiarizing persons with deafness with the digital environment. However, it should be noted that the measured health literacy levels in this study may have been influenced by confounding factors such as older age and comorbid conditions. Nonetheless, this study serves as a foundational contribution to the understanding of health literacy among persons with deafness with HTN in South Korea. Additionally, it provides valuable insights to guide the development of HTN self-management interventions for persons with deafness.
